# Effect of Viewing Video Representation of the Urban Environment and Forest Environment on Mood and Level of Procrastination

**DOI:** 10.3390/ijerph17145109

**Published:** 2020-07-15

**Authors:** Ernest Bielinis, Jenni Simkin, Pasi Puttonen, Liisa Tyrväinen

**Affiliations:** 1 Department of Forestry and Forest Ecology, Faculty of Environmental Management and Agriculture, University of Warmia and Mazury, Pl. Łódzki 2, 10-727 Olsztyn, Poland; 2 Natural Resources Institute Finland, Latokartanonkaari 9, 00790 Helsinki, Finland; jenni.simkin@luke.fi (J.S.); liisa.tyrvainen@luke.fi (L.T.); 3 Department of Forest Sciences, University of Helsinki, Latokartanonkaari 7, 00014 Helsinki, Finland; pasi.puttonen@helsinki.fi

**Keywords:** forest health benefits, forest therapy, procrastination, profile of mood states, pure procrastination scale, restorative outcome scale, subjective vitality scale, virtual nature

## Abstract

A common problem among students is the problem of delaying important work activities, which is conceptualized as procrastination. Since procrastination can cause considerable costs for society, we would like to find a method to effectively alleviate the symptoms of this conditioning. It has been proven in an earlier study that staying in the forest environment increases vitality and reduces anxiety, and the negative state of these features can be associated with the intensification of procrastination symptoms. Therefore, it is likely that watching a forest video may decrease the probability or intensity of procrastination. To measure the impact of the forest environment on the level of procrastination of the subjects, a randomized experiment was carried out, in which the subjects watched in random order (on different days) one of two 15-min videos: one showing a walk in the forest area and one showing a walk in an urban environment (control). We measured the level of so-called ‘fluid procrastination’ including three aspects: ‘lack of energy to do the work’, ‘inability to get to work’ and ’pessimistic attitude to do the work’ with a set of questions the respondents completed before and after the experiment. The results showed that one aspect of fluid procrastination (‘pessimistic attitude to do the work’) can be effectively lowered by watching a video showing the forest environment. In contrast, watching a video of an urban environment increased the procrastination levels for two other aspects of procrastination (‘lack of energy to do the work’, ‘inability to get to work’). We also measured three other parameters before and after the experiment: mood state, restoration and vitality. Watching the video from forest area raised mood and restoration and watching the video from urban area, decreased mood, vitality and restoration. The study suggests that watching a video showing forest landscapes could be used as an effective remedy for problems related to procrastination among students.

## 1. Introduction

### 1.1. Nature and Human Health

An increased amount of research has been carried out indicating the positive contribution of contact with nature to human health [1,2,3,4,5]. Since the 1990s, a growing number of studies have been focused on a comparison of natural and built-up urban environments [6,7,8] showing mostly beneficial effects of the natural environment for human health and well-being. There are several studies indicating the effectiveness of forest visits (in comparison to a build-up environment) on mental health, depression and anxiety among people [9,10,11,12]. There is also evidence that a short walk in a forest environment might also have a therapeutic impact on mental hospital patients with affective and psychotic disorders [13]. The treatment of various mental health problems can be supported by nature-assisted therapies such as hortitherapy and nature-assisted therapies [14]. It has been demonstrated that patients felt better when participating in programs consisting of planting and caring for plants [15,16]. The literature also describes forest therapy programs and their beneficial effects on the health of respondents [13,17]. Japanese and Korean research carried out in the field of the impact of the natural environment on human health is comprehensive [18,19,20] including research on how the forests environment impacts on human health [7,21,22].

Forest visits are demonstrated to have positive effects on human psychological relaxation [23], mental health [24] and the physical health [25]. According to study of Martens et al., (2011) walking in an urban forest environment has a positive effect on well-being [26], whereas Mitchell [27] showed that visiting the natural environment regularly, is associated with about a 6% lower risk of poor mental health. A walk in a forest with a therapist, as a form of forest therapy, had also a significant positive influence on the mental health of mental hospital patients and a session of around two hours was enough to obtain mental health benefits [13]. In addition, ten minutes of sitting or walking in a natural environment was enough to improve the mental health of college-aged students [28]. Furthermore, in a study in which a natural forest was viewed by participants in a virtual reality experiment, a session of six minutes of viewing was enough to support mental health [28]. Summing up, to support mental health, a subject’s contact with nature is important and can help providing good mental health.

In Finland, where the experiment was conducted, depression is a common problem, as in other European countries [29]. For example, in a study conducted in Eastern Finland, more than 11% of randomly-selected middle-aged people struggled with this problem [30]. A similar situation applies to students in Central Finland, where up to 14% of randomly selected respondents experience depression, and almost 28% experience psychological distress [31]. Moreover, procrastination can also be a significant problem, as it generally affects 15–20% of adults [32]. Procrastination is positively correlated with psychological distress [33] and therefore, finding activities and approaches that can help deal with procrastination would be valuable. The costs of mental health problems in EU countries can be estimated by the costs of their treatment within health care systems and social security costs, as well as through lower worker productivity and lost working time. In EU countries, these costs are equal to more than 4% of GDP across EU countries (over EUR 600 billion per year) [29]. In the context of health-promoting forest visits, it is worth mentioning that more than 70% of Finland’s land area is covered by forest areas, so the country has good accessibility to forests and the possibility of using these areas for promoting well-being [34]. The effectiveness of the impact of staying in a forest environment on improving mental well-being and reducing stress levels was demonstrated in Finland [35,36].

Five mechanisms have been identified [37] for explaining why visiting a forest effectively affects human health. There are some theories explaining the reasons for a positive human response to the natural environment through evolutionary psychology. Two of these theories are most commonly used. The first theory [38], the stress reduction theory (SRT), says that exposure to natural stimuli triggers a response of the parasympathetic nervous system leading to enhanced wellbeing. The measures used in this context are physiological (e.g., cortisol) as well as psychological measures [39]. The second theory, attention restoration theory (ART) [40,41,42], assumes that there are two types of attention, directed and involuntary attention. The ability to concentrate and use directed attention requires a cognitive effort, which can cause fatigue. Directed attention can be restored when involuntary attention is involved. Involuntary attention is facilitated in natural environments, which can help to restore directed attention and lead to improvements in cognitive functioning [43]. The level of restoration can be measured, for example, using the restorative outcome scale (ROS) questionnaire or perceived restoration scale (PRS) [44,45,46,47], which allows you to measure the result that is the hypothetic effect of ‘regenerating’ involuntary attention. The integrative approach in these two theories (SRT and ART) was developed by Kaplan [40]. In the model of combining these theories proposed by the author, insufficient attention resources will often be an antecedent of stress and both theories involve the possibility of restoring a person from a state of fatigue or stress to an optimal state. In summary, nature, including the forest environment, has the potential to regenerate mental strength and help returning to the optimal state after experiencing stress [48].

### 1.2. Virtual Nature Environments

To measure the positive health effects the forest environment has on humans, various methods have been used. The direct impact of the forest environment can be measured by staying in the forest. For this purpose, the simulation of various activities is used: using the forest environment during the camp [49], walking [9] or sitting and relaxing [17]. Control is often used, such as being in an urban environment, where a negative effect on the psychological and physiological well-being of the subjects has been observed [50]. Another way to assess the impact of the forest environment on humans is to simulate the impact of this environment in controlled conditions, for example by reproducing the images and sounds that occur in a forest environment, using a monitor [51], or by using virtual reality [52]. In this case, the control environment can be an image, a sound or virtual reality depicting the urban environment. Measurement in laboratory conditions allows better control over weather conditions because the measurements are carried out in relatively constant light conditions, sound intensity, temperature and humidity. In addition, the image and sound quality are always the same, so it is possible to reproduce the same conditions of forest and urban environments to any number of study participants.

Representing nature in the form of images, and video or virtual reality has been found to have a positive effect on human well-being, mental and physiological health [52,53,54]. Virtual nature (VN) environments reduces stress [55] and have a positive effect on dementia symptoms [56]. A virtual natural environment is a computer-generated form of reality which is similar to nature occurring in the real world [57]. Virtual nature environments can be described as a surrogate to real environments that can be used in situations, where authentic nature is not easily accessible. Showing photographs and/or videos have been the most common ways to study human response and the restorative potential of natural environments [58]. There are various forms of virtual reality, one of them is the ability to simulate the natural environment in the form of a film with sound and image from a natural area played on the screen and displayed to users [57]. Virtual forest nature had a significant influence on psychological parameters of participants in comparison with an urban environment [59]. Inspired by these results, a multisensory stimulation (video + sound) was used in this study with adaptation to the study of Pilotti et al. [58] in order to produce the expected effect in the respondents.

### 1.3. Nature in the Treatment of Procrastination and Measuring This Effect

As health benefits of nature are increasingly understood, it is worth paying attention to its possible use as a tool for helping people with difficulties in carrying out tasks. One of the problems individuals face every day is a problem doing tasks on time or delaying them, which results in various types of problems. This type of behavior, consisting of non-compliance with deadlines for psychological reasons, is called procrastination [60]. The procrastination problem can have a large global impact, when converted into a loss of money. The calculations made in the USA show that the procrastination is valued at a loss of $ 650 billion a year [61]. Procrastination especially affects students, causing difficulties in completing their tasks and eventually falling behind in their studies. Finding an effective method to help cope with this type of behavior would be very valuable [62].

Procrastination is often associated with anxiety [63], and sometimes also lower self-esteem [30]. Exposure to the natural environment, including the forest environment, which is useful in combating many other psychological ailments, could be a good way to influence the level of student procrastination. For example, fear anxiety or depression can be reduced by participating in a walk in a forest environment [13]. Therefore, this experiment was conducted by measuring the procrastination level of the subjects when watching videos of a forest and a city walk.

General procrastination can be measured with a variety of tools. The tool, that is validated and measures accurately procrastination, is pure procrastination scale (PPS) [64]. However, the PPS does not measure the change of procrastination at a current moment. In other words, it does not measure the fluid procrastination. If one wanted to measure the effect of visiting a forest environment on procrastination (on the level of general procrastination), an experiment would have to last for some time and the subjects would have to visit the forest regularly during this period (to determine whether their level of procrastination changed under the influence of these sessions). It would be difficult to measure general procrastination referring to the extensive and general life experiences of the respondents because the experiment in which the effect of any treatment is observed is only for a short time. We propose to conceptualize problems with timely performance, as the concept of ‘fluid procrastination’, which allows to measure the procrastination occurring ‘at a current moment’. Therefore, it is possible to measure the effects of short-term exposure to the forest environment on the level of fluid procrastination, because the value of this feature may change in a relatively short time. If fluid procrastination actually measures the level of procrastination at a given time, it will correlate with general procrastination PPS. In this study, it would be ideal if fluid procrastination correlates with PPS in some way, which again, would indicate that procrastination measured at a given time, would be higher in persons who tend to procrastinate generally. Other parameters can also be measured at the moment, such as mood, vitality or restorative properties, which is useful when we want to check the impact of videos from urban and forest areas on the participants. For the purposes of this study, we have defined a new type of procrastination, the fluid procrastination, which is a tendency for dysfunctional delay of tasks felt by a given person at the current moment. This can be measured using the ‘fluid procrastination scale’ (FPS).

It is also worth noting that by conducting the study with virtual videos, the method also allows assessing the suitability of not only the forest environment itself but also the video itself for reducing procrastination. If an individual would like to lower the level of procrastination, and the video would be useful for that, then it was enough for the individual to watch the video at home if a real forest environment is not easily accessible. Also, the key point is how to get nature exposure during work or studying, when participants need to get something done.

### 1.4. The Aim of the Research and Working Hypotheses

The aim of this research is to study the restorative effects and current procrastination of videos presenting forest and urban environments on Finnish university students.

A randomized controlled study was conducted, in which the subjects watched two types of video (a video showing a walk in a forest environment and a video showing a walk in an urban environment) with questionnaires measuring the fluid procrastination and other features filled before and after the watching.

The purpose of the study described here was to test three hypotheses:

**Hypothesis** **1.**  *(related to the research methodology)*: *Fluid procrastination will correlate with general procrastination. This will be manifested in the fact that people achieving higher values in the general procrastination questionnaire will also achieve higher fluid procrastination values*.

**Hypothesis** **2.**  *Fluid procrastination is a measurable feature which value may change under the influence of stimulation of the subjects in the form of displaying a video film of a walk in an urban or forest environment. It is hypothesized that the forest video decreases more the procrastination than the urban video*.

To check if the forest environment has the potential to reduce the level of procrastination, an experiment is carried out, in which the forest environment is presented in the form of a video to the subject and the fluid procrastination is measured before and after watching the video.

**Hypothesis** **3.**  *Values achieved by study participants on other psychological scales (profile of moods states, restorative outcome scale, subjective vitality scale), commonly used in forest well-being research after watching the video, will be similar to the results obtained on the fluid procrastination scale*.

In this study, we use three different scales to measure changes in mental well-being: profile of moods states (POMS), restorative outcome scale (ROS) and subjective vitality scale (SVS). Since these questionnaires allow measuring significant changes that are observed after exposure to the forest environment [65], therefore they can be used in this study as sensitivity indicators to measure the effect of displayed video on the subjects.

## 2. Materials and Methods

### 2.1. Subjects

The study participants were 42 healthy volunteer students (23 women, 19 men), with an average age of 26.24 ± 6.23 (mean ± SD) years. Of these, 40 subjects studied at the University of Helsinki, and two studied at the University of Turku. We aimed at recruiting students that did not have a professional relationship with nature through their studies. None of the students participating in the study studied biology, forestry or landscape design. All of the respondents lived in cities, the majority in the Helsinki Metropolitan area (39 respondents), and three outside it. Students were recruited to this experiment in the city center in a building (Porthania) belonging to the University of Helsinki. The recruiters (18 students participating in the ‘Urban Forestry’ course and studying at the University of Helsinki) explained to the respondents what the study was about. If the students were interested in participating in the experiment, they were given a recruitment card with information about the study and with specified recruitment criteria. The title of the study was ‘Supporting student well-being in everyday life’.

The recruiters explained that the respondents would be invited to two meetings, during which they would be presented with two videos (one 15-min video at one meeting). During the recruitment, it was explained that participants should complete a questionnaire regarding their well-being and also explained that both visits would take about half an hour.

To participate in the study, a participant needed to meet the following criteria (listed on the recruitment card handed to subjects): (1) had to agree to participate in these study and sign a written consent; (2) having an age between 18 and 44 years old; (3) does not take any medication that may affect mood and feelings; (4) had to give consent to treat personal data confidentially and for research purposes only; (5) does not consume large amounts of alcohol or other drugs that may affect them on examination days; (6) had to know Finnish (the questionnaires were prepared in Finnish). If recruited students answered ‘no’ to any of the above questions, they could not take part in the study. The responses were anonymous. Recruitment was conducted during 10 days from 30 September 2019 to 11 October 2019 (on workdays).

To determine the statistical power of the experiment, a statistical power analysis was performed using the free software ‘G * Power 3.1’ (Heinrich Heine University of Düsseldorf, Düsseldorf, Germany). Actual power (1-β error probability) for this research design was 0.977, assuming the presence of two groups and four repetitions, test with repeated measurements (two within-subjects factors), type of power analysis ‘Post hoc: Compute achieved power—given α, sample size, and effect size‘. The effect size was set at 0.25, α error of probability is 0.05. The number of 42 study participants was sufficient to identify significant differences between the compared levels.

All data were processed anonymously and protected in accordance with the guidelines of the EU General Data Protection Regulation 2016/679 (GDPR). Personal data was kept separate from the material being analyzed. Personal data was removed from the data when it is no longer needed for data linking. This study was positively evaluated by the Ethical Review Board at the University of Warmia and Mazury in Olsztyn. The number of the ethical statement is 07/2019. All the procedures were performed in this study in accordance with the ethical standards of the Polish Committee of Ethics in Science and with the 1964 Helsinki Declaration’s later amendments. All research was carried out in accordance with the recommendations of the University of Helsinki Ethics Committee.

### 2.2. Experimental Stimuli

Each subject watched a 15-min video showing a walk in the forest area and a 15-min video showing a walk in the urban area displayed in a room in the Porthania building.

Helsinki Central Park was chosen to make a video of the walk in the forest. It is a 10 km^2^ park that is popular among Helsinki residents. The Kruunuvuorenranta area of 1.42 km^2^ was also selected for recording in the forest environment. Three videos were used to make the video, the first video in the 15-min clip (0′:00″–4′:21″) showed a video shot in the Kruunuvuorenranta area (northern Helsinki). The video was shot in an area with canopy layer of a forest area with mature Scots pine (*Pinus sylvestris*) stands with some spruce and leafy understorey. The second video (4′:22″–8′:27″) was shot in the northern part of Helsinki Central Park, an area covered mature coniferous forests dominated by Scots pine and Norwegian spruce. Deciduous tree species and shrubs were found in the undergrowth. The third video (8′:28″–14′:58″) was shot in the southeastern part of Helsinki Central Park showing a forest walk on a path made of wooden beams. The walk led through a mature spruce stand with an admixture of birch and pine. The video shows picturesque landscapes enriched by lush developed undergrowth in this area.

To create a video showing the walk in the urban environment, the suburb areas in the Rastila and Vuosaari areas located in East-Helsinki were selected. Altogether four videos were shot in these areas, two were near the metro stations ‘Rastila’ and ‘Vuosaari’ (0′:00″–5′:17″), (9′:55″–12′:50″) and two in the eastern part of the suburbs (5′:18″–9′:54″), (12′:51″–15′:00″). In addition, to depict the view of buildings and streets, the video contained elements of normal, everyday life in this area: people, passing subways and cars. The footage in the video also includes urban greenery, lawns, and small-sized trees and shrubs, e.g., lime trees (*Tillia*).

The entire 15-min clips presented a real experience that is available in the administrative area of the city of Helsinki and can be repeated by a walk of anyone in this environment.

**Figure 1:**
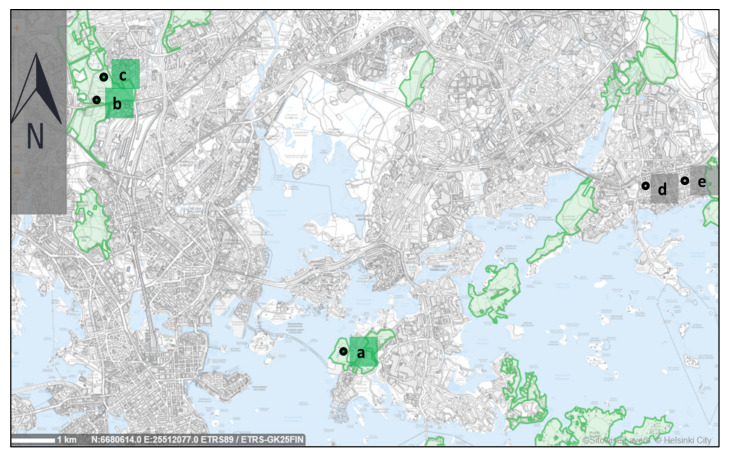
Map view of the part of the city of Helsinki with larger forest areas marked (light green fields) and marked places where the video for the experiment was made: ‘**a**’—place where the first video was taken from the forest area (Kruunuvuorenranta region); ‘**b**’—place of making the second video with the forest area, the northern part of the Central Park in Helsinki; ‘**c**’—location of the third video with forest area, the southern part of the Central Park in Helsinki; ‘**d**’—location of the first and third video from the urban area; ‘**e**’—the place of making the second and fourth video from the urban area. Map source: Helsinki map service, https://kartta.hel.fi (with own modifications).

**Figure 2:**
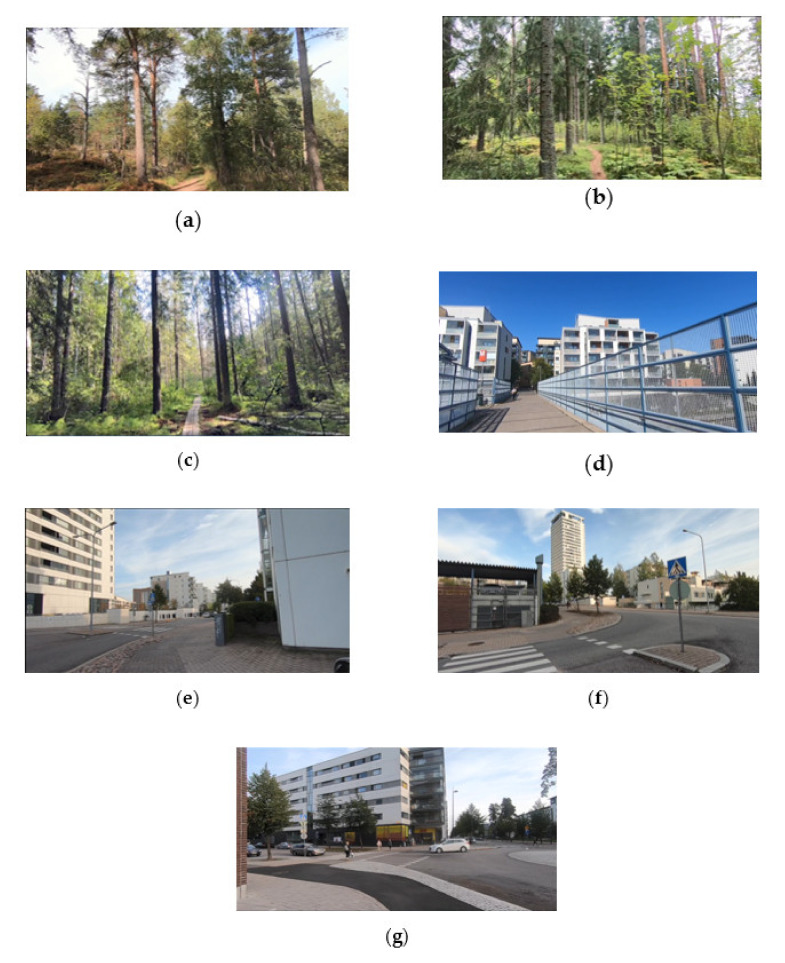
Shots from two videos displayed to study participants: (**a**) a shot from the first part of the video showing a walk in the forest (0′:00″–4′:22″); (**b**) a shot from the second part of the video of a walk in the forest (4′:22″–8′:27″); (**c**) a shot from the third part of the video on a walk in the forest (8′:28″–14′:58″); (**d**) a shot from the first part of the video showing a walk in the city (0′:00″–5′:17″); (**e**) a shot from the second part of the video of a walk in the city (5′:18″–9′:54″); (**f**) a shot from the third part of the video showing a walk in the city (9′:55″–12′:50″); (**g**) a shot from the fourth part of the video showing a walk in the city (12′:51″–15′:00″).

The videos were made in September 2019, at the turn of summer and autumn (the leaves were on the trees when preparing the videos). Areas of the city of Helsinki in which videos from the forest and urban area were shot are marked on the map (Figure 1). Shots from videos that make up the 15-min videos are shown in Figure 2.

All videos were prepared in HD quality (1440 px, 60 fps, with image stabilization), using a GoPro Hero 7 Black field camera (GoPro, Inc., San Mateo, CA, USA) and using the image stabilizing gimbal during filming movement (G5 3-axis Gimbal, FeiyuTech Co. Ltd., Guilin, China). During filming, the camera was kept at the operator’s eye level to recreate the experience as similar as possible to a walk in a forest or urban area. All videos were assembled using the iMovie application available for Mac OS (Apple Corp., Cupertino, CA, USA). To maintain satisfactory sound quality in the assembled video, commercially available soundtracks from soundsnap.com were used with high quality soundtracks recorded in forest and urban areas. The video finally selected for presentation to the subjects was selected in a multi-stage process, during which the assembled clips that most closely resembled normal daily experience were selected.

**Figure 3:**
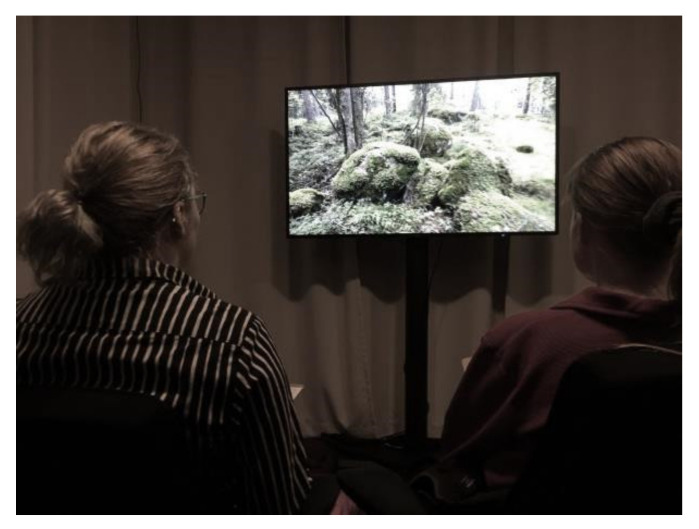
Participants in the study watching the displayed video (during experimental stimulation).

The videos were displayed to the subjects in the room using a high-resolution 55 “LCD monitor (MultiSync P554, NEC, Tokyo, Japan) with size of 71.4 × 124.4 cm^2^ (1920 × 1080 resolution). The experimental room was built on a rectangular plan with dimensions of 370 × 345 cm. The study participants sat on comfortable, adjustable chairs at a distance of 250 cm from the screen. There were a maximum of five people in the room during the experiment on separate, comfortable armchairs with back and shoulder rests. The volume of the loudspeakers associated with the monitor during the display of the screen was the same for the video from the forest area as well as for the video from the urban area, but the volume of the sound depended on the type of video and thus, the volume of the film from the urban area was higher than the volume of the film from the forest area. The view of the experimental room during the experimental stimulation is shown in Figure 3.

### 2.3. Procedure

Because the aim of the study was to check how videos depicting two different environments affect the fluid procrastination of subjects, a randomized experiment was performed during which videos were used as experimental stimulation. After signing consent to participate in the study (recruitment card), the participants were randomly assigned to the time and date on which they were to watch the videos. The video display order was random, simple randomization was used for this by assigning a random number to each hour and day of display. The principle of participation in the experiment was that each study participant had to watch each video once, and the minimum time before watching the second video should be 24 h. Based on these principles, the recruiters established a schedule that contained information about when a person would come to watch one of these two videos. The subjects were sent an e-mail reminder the day before the scheduled video and a text message on the day of experimental stimulation. 44 people were recruited to see each of the two videos once, but two did not appear to watch the second video. On each day of the experiment, three videos were shown at different times in the room in the Porthania building, at 13:00, at 14:30 and at 16:00. The display order of the two videos was randomized. To balance the video display effect (for the first or second time), it was attempted to see the video in a random way, first or second (counterbalancing).

**Figure 4:**
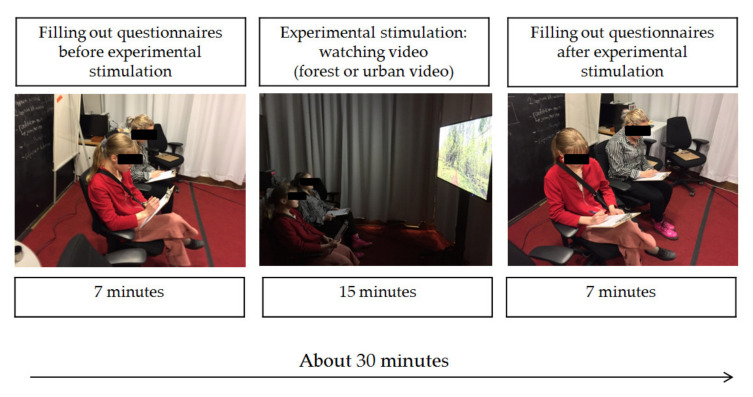
Illustration of the research process during the experiment.

On the first day, before watching the video, each participant completed an extended questionnaire, which contained general information on the characteristics of the subject and his/her previous experiences related to visiting green areas. The participants were also asked to fill in the Pure Procrastination Scale (PPS), which measured the level of general procrastination. In addition, participants completed a questionnaire containing four sets of questions with psychological scales before viewing: fluid procrastination scale (with three subscales), profile of mood states (POMS), restorative outcome scale (ROS) and subjective vitality scale (SVS). These measures were conducted just before watching the video and were called ‘Pre’ measures that measured participants’ current psychological state. The subjects then watched either an urban or forest video for 15 min (experimental stimulation), and after the video the subjects completed another questionnaire, ‘Post’. On the other date, the person filled out the ‘Pre’ questionnaire again, then watched another video, after which the person filled out the ‘Post’ questionnaire. The course of the experiment on one experimental day is shown in Figure 4. Since the purpose of this work was to present the results of the impact of virtual forest and urban environments on the level of fluid procrastination, mood, restoration and vitality other additional data (e.g., values of general procrastination, correlations of measured parameters with the data contained in questionnaire number one) are not published in this paper.

### 2.4. Measurements

The first time (regardless of whether it was a forest or city video), the examined person received an extended questionnaire. In the extended questionnaire, the respondents answered their daily experiences and questions that characterized themselves. In addition to standard questions about age, sex, place of residence and place of studies, the following questions were asked:

- Which of the following best describes your childhood (age under 16) environment? Circle the most appropriate number: from ‘1 = city center’ to ‘3 = rural’.
- How familiar are you with outdoor activities conducted in the woods? Circle the most appropriate number: from: 1 =‘not at all familiar’ to 5 = ‘very familiar’.
- How often do you go outdoors/in nature (park, forest, meadow, etc.) Circle the most appropriate number: May-September from: 1= ‘never’ to 5 = ‘5 times a week or more’.
- How important do you feel that the residence is a half-mile radius close to one of the green- or nature area? Circle the most appropriate number: from 1 = ‘Not important’ to 5 = ‘important’.

In addition, the background questionnaire contained the Finnish version of the pure procrastination scale (PPS), which makes it possible to measure the overall level of procrastination in the examined person. Procrastination is measured using questions relating to the life of the examined person or a certain period in that person’s life, which conceptualizes the procrastination as dysfunctional delay. The questions are for example ‘I am continually saying I’ll do it tomorrow’ or ‘I delay making decisions until it’s too late’. The original version of the questionnaire was developed by Steel [64], from which the Finnish version used in this study was validated among European validation studies [66]. The scale contains 12 items, each rated on a 5-point Likert scale. None of the items are reverse scored. Another example-item on the scale is ‘Putting things off till the last minute has cost me money in the past’ (1 = does not describe me at all, 5 = describes me very often or always). The Cronbach’s α value in international studies for this scale was between 0.89 and 0.93. Besides the PPS, each study participant completed the fluid procrastination scale (FPS), profile of mood states (POMS), restorative outcome scale (ROS) and subjective vitality scale (SVS) four times (Pre x 2 and Post x 2).

The FPS scale was used for the first time in this study. The formulation of FPS scale was based on the assumption that procrastination can also be measured at the current moment. Therefore, 11 statements were proposed, which the respondents rated on a 5-point Likert scale (1 = strongly disagree, 5 = strongly agree). The statements were ranked in three proposed subscales, which measures three separate aspects: (1) On scale 1 (FPS1) ‘lack of energy to do the work’ containing 4 items of which were reversed when calculating the results: ‘I feel right now that I would be good at meeting deadlines’, ‘Now I could immediately start doing something that is approaching a deadline’, ‘I feel that I will be able to complete my tasks’ and ‘I feel ready to do tasks’; (2) on scale 2 (FPS2) ‘inability to get to work’ containing 4 items: ‘I cannot do my tasks right now’, ‘I feel that now I could not make a decision and I would waste time on trivial matters’, ‘I feel discomfort at the very thought of doing something’, ‘I feel that it would be best to say right now: “I will do it tomorrow!”; (3) on scale 3 (FPS 3) ‘pessimistic attitude to do the work‘ containing 3 items: ‘At the moment I am worried about my future’, ‘At the moment I feel like I would not complete any task on time‘ and ‘I feel that I will lose something because of my lack of motivation to perform tasks’. It was assumed that these scales can measure the psychological effect faced by persons experiencing procrastination but as a result of own analysis, it was found that these three scales can significantly reflect the actual ruminations occurring in people with a tendency to delay the implementation of tasks. Unpublished pilot studies identified three main factors corresponding to the sub-scales of fluid procrastination.

The creation of the FPS scale was initiated in the pilot study launched before starting the actual field experiment. During these tests, questionnaire study was conducted in a room using a film from the urban and forest environment. The data was used to conduct exploratory factor analysis, during which, the statements based on factors were formulated and selected and to perform three subscales of the FPS scale. The subscales were named as presented already above ‘the lack of energy to do the work’, ‘inability to get to work’, ‘pessimistic attitude to do the work.’ When creating a group of items for potential use on an FPS scale, the practical significance of the items was assessed. To establish the FPS scale, only those statements were used that best correlated with the general procrastination and changed the most under the influence of viewing the video of urban and forest environment in the pilot study.

The impact of video on the mood of the subjects was also measured using the POMS scale. The original scale contains 65 items [67]. The scale in the Finnish version [68] contains 38 items and allows one to measure eight mood states: tension (four items, Cronbach’s α = 0.839), fatigue (three items, Cronbach’s α = 0.817), forgetfulness (four items, Cronbach’s α = 0.738), vigor (six items, Cronbach’s α = 0.882), depression (seven items, Cronbach’s α = 0.846), irritation (seven items, Cronbach’s α = 0.918), slackening (three items, Cronbach’s α = 0.753) and insecurity (five items, Cronbach’s α = 0.728). Each of the 38 statements was evaluated on a 5-point Likert scale (1 = not at all, 5 = completely).

The study also used the six-item ROS questionnaire [69] to assess the effect of video on the restorative effect on subjects. Each respondent assessed the items on a 7-point Likert scale (1 = not at all, 7 = totally). An example of an item that was used on a scale is ‘I feel restored and relaxed’ or ‘I feel focused and alert’. The value of Cronbach’s α for the scale in the experiment was 0.888.

The SVS questionnaire was also used to assess the level of vitality of the subjects after watching the video [70]. The scale consists of 4 items, which are assessed by respondents on a 7-point Likert scale. An example item is ‘I feel alive and vital’. The Cronbach’s α value for the scale in the experiment was 0.866.

The quality of the presented video was evaluated by subjects on a 5-point scale with two questions: ‘How pleasant the view of the displayed video was?’ and ‘How pleasant the sound of the displayed video was?’ However, many of the respondents understood the question differently than we had originally planned. They thought we asked about the content and not the quality of the videos, therefore we could not use these answers in our analysis.

The FPS scale was invented in this study and the analysis of its reliability and validity are good, and its usefulness has been proven here. The POMS, ROS and SVS scales are widely used in research on the impact of nature on subjects, and these scales are widely recognized as valid and reliable.

### 2.5. Data Analysis

Data are presented in the analyses as mean ± standard deviation (S.D.). Data analysis ‘variable by variable’ was adopted [71]. Raw data from questionnaires was used for the analysis. The two way-repeated measures of analysis of variance (ANOVA) was used, in which two within-subject factors were distinguished: conditioning (urban or forest video) and time (pre vs post). The interaction effect was also taken into account: conditioning × time. After the analysis of variance, post-hoc tests were calculated, Tukey’s test was used, and a *p* value < 0.05 was considered significant. The analysis was performed in the SPSS Statistics Version 25 program (IBM Corp., Armonk, NY, USA) and in the JMP 15 program (SAS Institute Inc., Cary, NC, USA). During the analysis, it was noted that some missing data appeared which were replaced by matched variables using the winsorization technique [72]. Also, the Expectation Maximization method, available in the SPSS package, was used. The data was also checked for unexpected values and the outliers were identified using the SPSS package and up to two outliers per group were identified. For each analysis, we calculated effect size ‘*η*^2^’: small = 0.01; medium = 0.06; and large = 0.14.

To check if there were correlations between subscales of fluid procrastinations scale and variables, a simple Pearson correlation coefficient was used. The results are summarized in Appendix A.

## 3. Results

### 3.1. Reliability and Validity of FPS Scale

The value of Cronbach’s α for the FPS1 subscale in the experiment was 0.869, for the FPS2 subscale 0.779 and for the FPS3 subscale (containing only three items) 0.676. Correlations of fluid procrastination subscales with the PPS scale were as follows (for *N* = 42): FPS1 *r* = 0.678 ***; FPS2 *r* = 0.600 ***; FPS3 *r* = 0.585 ***. Validity analysis of FPS is presented in the Table 1. The FPS scale is a new scale and the analysis of its reliability and validity are good, its usefulness has been proven here.

### 3.2. Fluid Procrastination Scale

The scale of fluid procrastination consists of three subscales (FPS1: Fluid procrastinations subscale—lack of energy to do the work; FPS2 fluid procrastinations subscale—inability to get to work; FPS3: fluid procrastinations subscale—pessimistic attitude to do the work) of which each subscale indicated a different response of this scale to the type of video displayed. Therefore, these scales were analyzed separately rather than one, total scale analysis. Two main factors were assessed in the statistical analysis of these subscales: the impact of experimental stimulation (conditions: forest video vs. urban video) and the impact of exposure to a given factor (time: pre vs. post). We were most interested in how the subscales change in time between the two environments, forest vs. urban. Therefore, also the interactions between conditioning and time were analyzed in the model. These effects and their interaction, calculated for the three subscales (FPS1, FPS2, FPS3) of fluid procrastination, were included in the two-way repeated measure ANOVA model. The results of these analyses are presented in Table 2. The only significant influence of the main factor ‘time’ on the studied subscales, was observed in the FPS3. The interaction between the time and conditioning was significant for all subscales of fluid procrastination, however, the effect size was only medium in these three cases (the value of η^2^ ranged from 0.06 to 0.14).

The mean values and standard deviations for each of the four measurement times (before forest video, after forest video, before urban video, after forest video) as well as the results of multiple comparisons by Tukey’s post-hoc test for the three subscales of fluid procrastination, are summarized in Table 3. In the case of the FPS1 subscale, the only significant difference was observed after watching a video depicting the urban environment compared to the situation before watching the video. This aspect of procrastination, ‘the lack of energy to do the work’, got worse by watching the urban video (*p* < 0.05, Urban: pre vs. post). In the case of the FPS2 subscale, the only significant difference was observed after watching a video depicting the urban environment compared to the situation before watching the video. This aspect of procrastination, ‘the inability to get to work’, got worse by watching the urban video (*p* < 0.05, Urban: pre vs. post). In the case of the FPS3 subscale, the value were significantly lower after watching a video of a walk in the forest landscape than before watching the video (*p* < 0.01, forest: pre vs. post) and also after watching the forest video compared to the city video (*p* < 0.05, post: urban vs. forest). This means that after watching a video of the forest environment, the aspect of procrastination ‘the pessimistic attitude to do the work’ lowered among participants.

### 3.3. Profile of Mood States

In the case of the POMS questionnaire, the main effects were also analyzed: conditions (forest vs. urban video) and time (pre vs. post) and the interaction of these effects. The results of the analysis of these factors and their interactions using the two-way repeated measure ANOVA model are presented in Table 4. For six out of eight analyzed subscales, the interaction between conditioning and time was significant (tension, fatigue, forgetfulness, vigor, irritation, slackening). Considering the main effects, Conditions had a significant impact on tension and insecurity, while Time had a significant impact on tension, depression, vigor and insecurity. The effect size for some variables in main and interaction effects was very high, sometimes even twice as large as the effect size of the procrastination subscales. The largest effect was observed in the case of the tension, vigor, irritation and slackening subscales.

The result of multiple comparisons with the Tukey test (Table 5) indicates that the values of tension, fatigue, forgetfulness and irritation significantly decreased after watching the video from the forest area (Forest: pre vs. post). The vigor value decreased significantly after watching a video from the urban area and the slackening value increased (urban: pre vs. post). There were no significant differences between the POMS subscale values when comparing pre-test results (pre: forest vs. urban). The values of tension, forgetfulness and slackening subscales were significantly higher after watching a video from the urban environment, compared to the video from the forest environment (post: urban vs. forest).

### 3.4. Restorative Outcome Scale and Subjective Vitality Scale

Scales ROS and SVS, are often used as good indicators of the effect of urban and forest environment on the subjects. They are often used together, so for the purposes of this description they are included in one chapter.

In the case of the ROS scale and the SVS scale, two-way repeated measure ANOVA was used to examine the effect of the conditioning effect and the time effect, as well as the interaction effect on these variables (Table 6). The conditioning effect was not significant for ROS or SVS but the Ttime effect was significant for the SVS scale. We were most interested in how the restoration and vitality change in time between the two environments, forest vs. urban. Therefore, the interactions between the conditioning and time were also analyzed in the model. The interaction effect was significant on both scales, ROS and SVS.

The results of the Tukey’s multiple comparison tests for the ROS and SVS are included in Table 7. In the case of the ROS, the restoration was significantly higher after watching the forest environment video than before watching it (forest: pre vs. post) while the SVS did not change significantly. Both, ROS and SVS decreased significantly from watching the urban video compared to the state before watching it (urban: pre vs. post). The values of ROS and SVS scales did not differ significantly between pre-tests (pre: forest vs. urban). The ROS value was significantly higher after watching the video from the forest environment than after watching the video from the urban environment, for the SVS scale value there were no such differences (post: urban vs. forest).

### 3.5. Comparison of Effect Size

It is worth noting that in the conducted experiment the size of the effect in the case of tested fluid procrastination was not large (the value of *η*^2^ for each of the three subscales was in the range of 0.06 to 0.14). Compared to the effect size of other measured variables (e.g., certain mood states or restoration)—the effect size was often two times smaller in the case of fluid procrastination.

## 4. Discussion

### 4.1. Fluid Procrastination

#### 4.1.1. The Impact of the Forest Environment on the Level of Procrastination

The impact of the forest environment (in this case: its representation in video form) on the level of procrastination has not been studied before. To examine this impact, a measurement of ‘fluid procrastination’ or procrastination ‘at the moment’ was proposed, which, as expected, changed after watching the urban and the forest video. This measurement was possible due to simulation in controlled conditions of walking in the forest environment and walking in the urban environment—as a control. The created fluid procrastination scale consists of three smaller scales. The values of all three scales significantly changed under the influence of watching videos showing two environments. Subscale 1 and subscale 2 values (FPS1: fluid procrastinations subscale—lack of energy to do the work; FPS2 fluid procrastinations subscale—inability to get to work) increased (got worse) significantly after exposure to video from the urban area.

This confirms Hypothesis number 1—the values of fluid procrastination may change under the influence of the subjects’ experience and can be changed by stimulation. This means that the view of the urban area significantly increased the temporary tendency of the respondents to procrastination. When going for a walk, to reduce the worry associated with procrastination, individual should prefer the forest environment. This might be helpful especially if an individual lacks a positive attitude towards work and also if an individual cannot get work started. Subscale value 3 (FPS3: fluid procrastination subscale—pessimistic attitude to do the work) decreased significantly after exposure to a video of a walk in the forest. This result is of great practical importance as when a person cannot get down to the task, watching a video showing the forest landscape might help. This walk (or video watched) should significantly reduce the aspect related to the occurrence of negative thinking, which has been suggested to be a key factor responsible for problems with the impossibility to get on with tasks [73]. The urban-forest area relation has also been repeatedly studied with some indication, that being in an urban area may adversely affect to the mental health of the subjects [74]. These findings are strengthened in comparison with the results presented in this study—viewing urban environment represented by a 15-min video—significantly reduced the level of energy to perform tasks and the ability to do work. These results also support Hypothesis 2 in this study, saying that video from the forest areas reduces short-term procrastination more, than the video from an urban area. Previous studies have shown that psychological well-being can be increased through the impact of the forest environment [43,65,75], including in a virtual form [59]. Current research has shown that fluid procrastination can also be changed by representing the natural environment in the form of a video film.

#### 4.1.2. Relationship between Procrastination and Short-Term Procrastination

Fluid procrastination in this study was significantly correlated with general procrastination, as expected. People achieving higher values in the general procrastination questionnaire, achieved higher values in the fluid procrastination subscales. However, correlation coefficients did not reach a value greater than *r* = 0.678, which means, that these subscales of procrastinations did correlate within each other, but it did not reach the total correlation. Therefore, the use of fluid procrastination as a measurement tool for ‘at the moment’, is only legitimate. Furthermore, due to significant correlation, it is highly probable that fluid procrastination measures the aspects of procrastination as a phenomenon. This is due to the fact that fluid procrastination ‘at the moment’ is higher in people with higher values of general procrastination. This confirms “Hypothesis number 1”—these two types of procrastination are related to each other.

### 4.2. Mood States

The interaction between conditioning and time was significant in case of six of the examined mood states: tension, fatigue, forgetfulness, vigor, irritation, slackening. After watching the video showing the forest area, the four subscales of the POMS questionnaire significantly decreased: tension, fatigue, forgetfulness and irritation. In the case of subscale 3 (FPS3: ‘pessimistic attitude to do the work’) of fluid procrastination, decreased values were also observed. Therefore it can be concluded that the reduction of the four mentioned mood subscales are associated with a decrease level of fluid procrastination. This result is consistent with the results of other studies, in which general procrastination was associated with negative mood changes [76]. The level of the vigor subscale did not increase after watching the video from the forest area, which might be due to the fact, that the study was conducted in the room and not in the actual nature. Without the active movement—it is simply an insufficiently stimulating environment to raise the level of vigor. However, in previous studies, the level of vigor did increase [59,77]. Watching a video from the urban environment, significantly reduced the level of vigor and slackening, which resembles the results obtained in a previous virtual reality experiment conducted in Taiwan [59]. The results of measurement of fluid procrastination indicate that in this set (urban: pre vs. post) two subscales significantly increased their values (FPS1 and FPS2), which means that these changes are associated with lowering vigor level and increasing slackening level. There were no differences between the POMS scale values in the case of two pre-tests, which indicates that the group was correctly randomized, and the value did not differ significantly in both groups. In the comparison of the two videos in the post-test, the difference was observed between the impact of the video from the forest area and the video from the urban area in the case of three mood states: tension, forgetfulness and slackening. In this compilation, the scale of fluid procrastination FPS3 reached significantly higher values after displaying the urban video. It can be assumed that a pessimistic attitude toward work may increase as the level of slackening increases and the level of vigor decreases. In many studies on forest therapy, it has been shown that the values of POMS subscales can change after exposure to forest areas in their various configurations [78,79,80,81,82]. The observed changes in the value of the POMS subscales logically change similarly to the value of the fluid procrastination scale subscales, which confirms ‘Hypothesis 3’. This hypothesis assumes that under the influence of the viewed videos, not only will the value of fluid procrastination change, but also the value of other instruments for measuring psychological features will change, in this case profile of mood states, which has been observed in many previous studies.

### 4.3. Restorative Outcome and Subjective Vitality

The impact of the forest environment (or in this case: its representation in video form) on the level of the ROS scale value was significant, which means that the presented video had a restorative effect on the subjects. A change under the influence of this video also occurred in the case of the subscale of FPSFPS1 fluid procrastination, which means that as restorative activity increased, pessimistic attitude to the work decreased. The SVS value did not increase significantly after watching a video of the forest area. This mirrors the results obtained for the POMS sub-scale “vigor’, which also did not change after watching the video from the forest area. After watching a video from the urban environment, both restorative and subjective vitality decreased. Of course, this is an unfavorable change, because it is desirable to increase the value of these features. A video from the urban environment turned out to be unsuitable for causing a restorative effect. As previous research has reported [83,84], the urban environment can negatively influence the psychological outcomes, which may have a negative effect on people living in the urban environment. The values of the ROS scale and the SVS scale did not differ significantly before the study (pre: forest vs. urban), which indicates the correct randomization of the study and the good balance of the research groups. In the case of comparing respondents’ responses after watching a video from the forest and urban environment (post: forest vs. urban), only the ROS scale values were significantly different. The ROS value was significantly higher after watching the video from the forest area in comparison to the urban area, while the SVS scale value did not differ significantly. For the subscale of fluid procrastination, the value of the FPS3 subscale was lower after watching a video from the forest environment, which means that as the restorative rate increased and the pessimistic attitude to work declined. This confirms Hypothesis number 3—when the values of ROS and SVS scales change, the values of the subscales of fluid procrastination also change, which is in line with the results of previous studies on forest recreation [65,85,86,87].

### 4.4. Future Research Directions

In future studies, more understanding is needed about the dose-response relationships (e.g., regular walks in the forest, staying at the camp in the forest [88,89]) of the repeated use of a real forest environment on the general level of procrastination on the subjects. These studies can be combined with an experiment during which, the videos of the forest environment will be displayed. It will then be possible to check if the effect caused by a video stimulation is as good as the natural experience in real environment.

Using glasses for virtual reality [90] may also be a good example of forest environment simulation and attempts to reduce procrastination. Participation in a virtual walk could be a good and easily available restorative method if its effectiveness was confirmed.

It is also possible to refine the fluid procrastination scale to make the measured effect larger, for which the sensitivity of the scale should be increased. However, the impact of the natural forest environment on the values of fluid procrastination may be much greater than the impact of the displayed video. This would indicate that a more effective form of dealing with procrastination would be a walk in a real forest environment, in which case the sensitivity of the scale would not have to be increased.

The content of the videos also reflected everyday environments for Helsinki residents. People may prefer different types of environments in their everyday life. For example, in the real natural environment, people with a stronger nature relatedness recover from stress more in the forest environment compared to the city center (e.g., [85]). In consequence, preferences for the content of virtual nature environments may also differ between individuals.

The significance of research results is great for societies living in cities. Contemporary society is struggling with the so-called ‘technostress’ [91] caused by the necessity of living among buildings, cars, in concrete buildings. We suggest that under these conditions, procrastination capacity may be greater. The recommendation resulting from our research is the proposal to use virtual reality representing the image of the forest to reduce the level of procrastination. The effectiveness of such action has been experimentally proven in the current research. In future research, the wider use of virtual reality representing forest areas for an urbanized society should be explored.

## 5. Conclusions

In this study, the effects of two types of video on fluid procrastination, mood, restorative and vitality in young adults studying at the university were examined. Participants in the study saw a video showing a walk in a forest environment and a video showing a walk in an urban environment (as a control). Both videos were recorded in an urban area of the city of Helsinki. These two videos were shown to participants (experimental stimulation). The psychological questionnaire was completed by the subjects before watching the video (pre-test) and after watching the video (post-test). The results indicate that a video showing a walk in the forest environment can effectively reduce one aspect of the measured fluid procrastination—‘pessimistic attitude to do the work’. This video can also be useful for lowering negative moods, such as tension, fatigue, forgetfulness, irritation or raising the level of restoration. On the other hand, watching video from an urban area can increase two aspects of measured procrastination—it can worsen the lack of energy to do the work and the level of inability to get to work. Watching a movie from the urban environment can also lower vigor, restoration and vitality, and increase slackening. The results of the study are promising and suggesting that to reduce the level of procrastination it is worth watching a video showing nature. In addition, the study used the concept of the scale of fluid procrastination, the use of which proved to be justified—the scale logically changed its values under the influence of watched videos and correlation with subscales and general procrastination was observed, the scale was also reliable (good Cronbach’s α coefficients).

## Figures and Tables

**Table 1:** Correlation results between fluid procrastination scale and fluid procrastination scale subscales (validity).

	FPS 1 (Items 1–4)	FPS 2 (Items: 5–8)	FPS 3 (Items 9–11)	FPS Total (Items: 1–11)
**FPS 1**	-			
**FPS 2**	0.733 ***	-		
**FPS 3**	0.668 ***	0.695 ***	-	
**FPS Total**	0.906 ***	0.908 ***	0.867 ***	-

*** Correlation is significant at the 0.001 level (2-tailed); FPS1—fluid procrastination scale subscale 1; FPS2—fluid procrastination scale subscale 2; FPS3—fluid procrastination scale subscale 3; FPS Total—fluid procrastination scale for all items included in the scale.

**Table 2:** Results of two-way repeated-measures ANOVA for the fluid procrastination scale (three subscales).

Fluid Procrastination Scale	Main Effect									Interaction			
	Conditioning				Time					Conditioning × Time			
	Forest vs. Urban				Pre vs. Post								
	*F*	*p*		*η* ^2^	*F*	*p*		*η* ^2^		*F*	*p*		*η* ^2^
FPS1	0.990	0.326	-	0.024	1.215	0.277	-	0.029	-	4.482	0.040	*	0.099
FPS2	1.547	0.221	-	0.036	3.020	0.090	-	0.069	-	4.642	0.037	*	0.102
FPS3	2.465	0.124	-	0.057	5.489	0.024	*	0.118	-	4.831	0.034	*	0.105

* Is significant at *p* < 0.05, - = Not significant, two-way repeated measure ANOVA; FPS1: Fluid Procrastinations Subscale—lack of energy to do the work; FPS2 Fluid Procrastinations Subscale—inability to get to work; FPS3: Fluid Procrastinations Subscale—pessimistic attitude to do the work; n = 42; Effect sizes (*η*^2^): 0.01 (small), 0.06 (medium) and 0.13 (large).

**Table 3:** Results of multiple comparisons of three fluid procrastination subscales for forest versus urban videos, as well as before and after exposure to videos.

	**Forest**						**Urban**					
	**Pre**		**Post**				**Pre**		**Post**			
	Mean	S.D.	Mean	S.D.	*p*		Mean	S.D.	Mean	S.D.	*p*	
FPS1	2.43	0.55	2.37	0.63	0.4792	-	2.40	0.76	2.60	0.74	0.0255	*
FPS2	2.35	0.63	2.32	0.73	0.9948	-	2.29	0.69	2.60	0.82	0.0425	*
FPS3	2.48	0.80	2.21	0.64	0.0021	**	2.47	0.82	2.49	0.87	0.7760	-
	**Pre**						**Post**					
	**Forest**		**Urban**				**Forest**		**Urban**			
	Mean	S.D.	Mean	S.D.	*p*		Mean	S.D.	Mean	S.D.	*p*	
FPS1	2.43	0.55	2.40	0.76	0.7921	-	2.37	0.63	2.60	0.74	0.0543	-
FPS2	2.35	0.63	2.29	0.69	0.9711	-	2.32	0.73	2.60	0.82	0.1054	-
FPS3	2.48	0.80	2.47	0.82	0.9421	-	2.21	0.64	2.49	0.87	0.0119	*

** Is significant at *p* < 0.01, * is significant at *p* < 0.05, - = Not significant, ANOVA-Tukey’s test; FPS1: Fluid Procrastinations Subscale—lack of energy to do the work; FPS2 Fluid Procrastinations Subscale—inability to get to work; FPS3: Fluid Procrastinations Subscale—pessimistic attitude to do the work; S.D.: Standard Deviation; n = 42.

**Table 4:** Results of two-way repeated measure ANOVA for Profile of Mood States.

POMS	Main Effect								Interaction			
	Conditioning: Forest vs. Urban				Time: Pre vs. Post				Conditioning × Time			
	*F*	*p*		*η* ^2^	*F*	*p*		*η* ^2^	*F*	*p*		*η* ^2^
Tension	6.232	0.017	*	0.132	11.507	0.002	**	0.219	15.145	0.0004	***	0.270
Fatigue	0.145	0.705	-	0.004	3.022	0.090	-	0.069	5.529	0.024	*	0.119
Forgetfulness	1.976	0.167	-	0.046	3.468	0.070	-	0.078	9.229	0.004	**	0.184
Vigor	0.110	0.742	-	0.003	6.356	0.016	*	0.134	11.227	0.002	**	0.215
Depression	1.324	0.257	-	0.031	7.691	0.008	***	0.158	2.755	0.105	-	0.063
Irritation	0.099	0.755	-	0.002	3.490	0.069	-	0.078	12.698	0.001	**	0.236
Slackening	1.675	0.203	-	0.039	0.067	0.797	-	0.002	10.781	0.002	**	0.208
Insecurity	8.311	0.006	**	0.169	9.569	0.004	**	0.189	2.807	0.102	-	0.064

*** Is significant at *p* < 0.001, ** is significant at: *p* < 0.01, * is significant at *p* < 0.05, - = Not significant, two-way repeated measure ANOVA; POMS: Profile of Mood States; n = 42; Effect sizes (*η*^2^): 0.01 (small), 0.06 (medium) and 0.13 (large).

**Table 5:** Results of multiple comparisons of Profile of Mood States scores for forest versus urban videos, as well as before and after video exposures.

	**Forest**						**Urban**					
	**Pre**		**Post**				**Pre**		**Post**			
	Mean	S.D.	Mean	S.D.	*p*		Mean	S.D.	Mean	S.D.	*p*	
Tension	1.78	0.62	1.29	0.43	<0.001	***	1.70	0.61	1.74	0.66	0.9805	-
Fatigue	2.59	0.92	2.27	0.87	0.0311	*	2.46	0.81	2.49	0.83	0.9871	-
Forgetfulness	2.02	0.76	1.72	0.62	0.0065	**	1.97	0.79	2.05	0.71	0.9980	-
Vigor	2.63	0.71	2.66	0.87	0.9913	-	2.85	0.77	2.38	0.74	0.0011	**
Depression	1.58	0.55	1.40	0.44	0.0159+	+	1.58	0.53	1.54	0.57	0.8486	-
Irritation	1.35	0.40	1.13	0.22	0.0026	**	1.22	0.30	1.28	0.44	0.7197	-
Slackening	1.85	0.73	1.72	0.78	0.1120	-	1.86	0.84	2.03	0.89	0.0450	*
Insecurity	1.70	0.55	1.44	0.42	0.0076+	+	1.78	0.59	1.70	0.47	0.6598	-
	**Pre**						**Post**					
	**Forest**		**Urban**				**Forest**		**Urban**			
	Mean	S.D.	Mean	S.D.	*p*		Mean	S.D.	Mean	S.D.	*p*	
Tension	1.78	0.62	1.70	0.61	0.8509	-	1.29	0.43	1.74	0.66	0.0003	***
Fatigue	2.59	0.92	2.46	0.81	0.7661	-	2.27	0.87	2.49	0.83	0.3848	-
Forgetfulness	2.02	0.76	1.97	0.79	0.9601	-	1.72	0.62	2.05	0.71	0.0337	*
Vigor	2.63	0.71	2.85	0.77	0.3004	-	2.66	0.87	2.38	0.74	0.1145	-
Depression	1.58	0.55	1.58	0.53	0.9995	-	1.40	0.44	1.54	0.57	0.2650	-
Irritation	1.35	0.40	1.22	0.30	0.2843	-	1.13	0.22	1.28	0.44	0.1100	-
Slackening	1.85	0.73	1.86	0.84	0.9169	-	1.72	0.78	2.03	0.89	0.0237	*
Insecurity	1.70	0.55	1.78	0.59	0.7137	-	1.44	0.42	1.70	0.47	0.0112+	+

*** Is significant at *p* < 0.001, ** Is significant at *p* < 0.01, * is significant at *p* < 0.05, - = Not significant, ANOVA-Tukey’s test; + Not significant in two-way repeated measure ANOVA; S.D.: Standard Deviation; n = 42.

**Table 6:** Results of two-way repeated measures ANOVA for restorative outcome scale and subjective vitality scale.

ROS and SVS	Main Effect								Interaction			
	Conditioning: Forest vs. Urban				Time: Pre vs. Post				Conditioning × Time			
	*F*	*p*		*η* ^2^	*F*	*p*		*η* ^2^	*F*	*p*		*η* ^2^
ROS	0.237	0.629	-	0.006	0.001	0.983	-	<0.0001	21.469	<0.001	***	0.344
SVS	0.029	0.865	-	0.001	4.093	0.0496	*	0.091	9.798	0.003	**	0.193

*** Is significant at: *p* < 0.001, ** is significant at *p* < 0.01, * is significant at *p* < 0.05, - = Not significant, two-way repeated measure ANOVA; ROS: restorative outcome scale; SVS: subjective vitality scale; n = 42; Effect sizes (*η*^2^): 0.01 (small), 0.06 (medium) and 0.13 (large).

**Table 7:** Results of multiple comparisons of restorative outcome scale and subjective vitality scale for forest versus urban videos, as well as before and after exposure to videos.

	**Forest**						**Urban**					
	**Pre**		**Post**				**Pre**		**Post**			
	Mean	S.D.	Mean	S.D.	*p*		Mean	S.D.	Mean	S.D.	*p*	
ROS	4.04	0.74	4.44	0.78	0.0181	*	4.38	0.97	3.98	0.98	0.0196	*
SVS	4.46	0.89	4.55	1.02	0.8778	-	4.70	1.10	4.27	1.11	0.0041	**
	**Pre**						**Post**					
	**Forest**		**Urban**				**Forest**		**Urban**			
	Mean	S.D.	Mean	S.D.	*p*		Mean	S.D.	Mean	S.D.	*p*	
ROS	4.04	0.74	4.38	0.97	0.0985	-	4.44	0.78	3.98	0.98	0.0153	*
SVS	4.46	0.89	4.70	1.10	0.4933	-	4.55	1.02	4.27	1.11	0.3276	-

** Is significant at *p* < 0.01, * is significant at *p* < 0.05, - = not significant, ANOVA-Tukey’s test; ROS: restorative outcome scale; SVS: subjective vitality scale; S.D.: standard deviation; n = 42.

## Appendix A

**Table A1:** Correlations (Pearson’s *r*) between three subscales of fluid procrastination scale and other traits measured by questionnaires in four different times: before viewing forest video (forest, pre), after viewing forest video (forest, post), before viewing urban video (urban, pre), after viewing urban video (urban, post).

	Forest						Urban					
	Pre			Post			Pre			Post		
	FPS1	FPS2	FPS3	FPS1	FPS2	FPS3	FPS1	FPS2	FPS3	FPS1	FPS2	FPS3
Gender	0.245	0.227	0.219	0.255	0.104	0.290	0.040	0.148	0.085	0.097	0.092	0.074
Age	−0.114	−0.168	−0.022	0.005	−0.040	−0.089	0.030	−0.077	−0.080	0.109	0.000	−0.066
Childhood environment	−0.066	0.021	0.177	−0.176	−0.124	0.049	0.166	0.067	0.143	0.294	0.240	0.275
Familiar with outdoor activities	−0.255	−0.142	−0.232	−0.077	0.033	−0.281	−0.216	−0.240	−0.174	−0.110	−0.038	−0.172
Outdoor frequencies	−0.267	−0.187	−0.192	−0.055	0.058	−0.265	0.032	−0.127	0.003	−0.056	−0.003	−0.036
Importance of green area neighborhood	−0.213	−0.136	−0.268	−0.282	−0.220	−0.440 **	−0.052	−0.120	−0.119	−0.018	−0.013	−0.148
POMS												
Tension	0.236	0.192	0.364 *	0.046	−0.066	−0.030	0.028	0.118	0.323 *	0.097	0.184	0.285
Fatigue	0.529 ***	0.506 **	0.446 **	0.455 **	0.384 *	0.273	0.413 **	0.441 **	0.533 ***	0.586 ***	0.431 **	0.416 **
Forgetfulness	0.518 ***	0.558 ***	0.533 ***	0.505 **	0.470 **	0.335 *	0.661 ***	0.622 ***	0.550 ***	0.622 ***	0.692 ***	0.615 ***
Vigor	−0.503 **	−0.579 ***	−0.373 *	−0.513 **	−0.519 ***	−0.205	−0.564 ***	−0.686 ***	−0.543 ***	−0.616 ***	−0.630 ***	−0.532 ***
Depression	0.283	0.355 *	0.450 **	0.102	0.103	0.398 **	0.512 **	0.519 ***	0.594 ***	0.401 **	0.501 **	0.565 ***
Irritation	0.267	0.123	0.238	0.035	0.027	0.041	0.144	0.130	0.313 *	0.200	0.385 *	0.360 *
Slackening	0.540 ***	0.631 ***	0.500 **	0.509 **	0.518 ***	0.461 **	0.695 **	0.670 ***	0.618 ***	0.611 ***	0.707 ***	0.600 ***
Insecurity	0.206	0.237	0.430 **	−0.119	0.018	0.332*	0.323 *	0.465 **	0.534 ***	0.208	0.441 **	0.399 **
ROS	−0.472 **	−0.557 ***	−0.501 **	−0.484 **	−0.497 **	−0.401 **	−0.618 ***	−0.631 ***	−0.732 ***	−0.723 ***	−0.771 ***	−0.683 ***
SVS	−0.569 ***	−0.613 ***	−0.519 ***	−0.572 ***	−0.536 ***	−0.433 **	−0.630 ***	−0.704 ***	−0.605 ***	−0.620 ***	−0.663 ***	−0.608 ***

***: *p* < 0.001, **: *p* < 0.01, * *p* < 0.05; FPS1: fluid procrastinations subscale – lack of energy to do the work; FPS2 fluid procrastinations subscale—inability to get to work; FPS3: fluid procrastinations subscale—pessimistic attitude to do the work; Gender: Gender (female-1, male-2); childhood environment: which of the following best describes your childhood (age under 16) environment? (from ‘1-city center’ is to ‘3-rural’); familiar with outdoor activities: how familiar are you with outdoor activities conducted in the woods? Circle the most appropriate number (answers: 1 = not at all familiar to 5 = very familiar); outdoor frequencies: how often do you go outdoors/in nature (park, forest, meadow, etc.) (May-September): 1 = never, 5 = 5 times a week or more; Importance of green area neighborhood: How important do you feel about it, that the residence is a half-mile radius close to a half-mile radius of one of the green- or nature area?: 1-Not important, 5-important; POMS: profile of mood states; ROS: restorative outcome scale; SVS: subjective vitality scale; n = 42.
